# Fixel-Based White Matter Correlates of Sentence Comprehension in Post-Stroke Aphasia

**DOI:** 10.3390/brainsci15101039

**Published:** 2025-09-25

**Authors:** Dongxiang Fang, Xiangtong Ji, Haozheng Li, Shuqi Xu, Yalan Yang, Jiayun Zhan, Anthony Pak-Hin Kong, Ruiping Hu

**Affiliations:** 1 Department of Rehabilitation Medicine, Huashan Hospital, Fudan University, Shanghai 200040, China; dongxiangfang@fudan.edu.cn (D.F.); ji_xiangtong@fudan.edu.cn (X.J.); lihaozheng3211@163.com (H.L.); 24211220065@m.fudan.edu.cn (S.X.); 2 Department of Rehabilitation Sciences, East China Normal University, Shanghai 200050, China; yangyalan_ecnu@163.com; 3 Kenneth P. Dietrich School of Arts and Sciences, University of Pittsburgh, Pittsburgh, PA 15260, USA; jiz450@pitt.edu; 4 Academic Unit of Human Communication, Learning and Development, The University of Hong Kong, Hong Kong SAR 999077, China; 5 The Aphasia Research and Therapy (ART) Laboratory, The University of Hong Kong, Hong Kong SAR 999077, China

**Keywords:** aphasia, sentence comprehension, diffusion MRI, fixel-based analysis

## Abstract

Background/Objectives: Auditory sentence comprehension often remains impaired in individuals with post-stroke aphasia despite recovery in word-level comprehension. Neuroimaging studies have identified a left perisylvian network, especially temporal regions, as central to sentence comprehension, while the role of left frontal areas and specific white matter tracts remains debated. This study uses advanced fixel-based analysis (FBA) of diffusion MRI to precisely map white matter alterations related to complex sentence comprehension deficits in subacute Mandarin-speaking aphasic patients, addressing gaps from prior voxel-based and English-specific research. Methods: Twenty-three right-handed native Mandarin speakers with subacute (1–6 months post-onset) single left-hemisphere strokes underwent diffusion MRI. Standard preprocessing and FBA were conducted. Whole-brain linear regression assessed associations between fiber density and cross-section (FDC) and non-canonical sentence comprehension, controlling for age, education, time post-stroke, and verb comprehension. Mean FDC was calculated for each tract containing at least one significant fixel identified by FBA. Partial Spearman’s correlations examined relationships between mean FDC values within these tracts and comprehension accuracy for each sentence type, controlling for the same covariates. Results: Canonical sentences were comprehended significantly better than non-canonical sentences. FBA identified significant positive correlations between FDC and non-canonical sentence comprehension in the left superior longitudinal fasciculus (SLF II and SLF III), arcuate fasciculus (AF), middle longitudinal fasciculus, inferior fronto-occipital fasciculus, and the isthmus and splenium of the corpus callosum. Fiber density reduction primarily drove reductions in FDC, whereas reductions in fiber cross-section were limited to dorsal tracts (SLF III and AF). Conclusions: This study highlights a distributed left perisylvian white matter network critical for complex sentence comprehension in Mandarin speakers, refining neurocognitive models by identifying specific white matter substrates and demonstrating FBA’s utility in aphasia research.

## 1. Introduction

The sentence is one of the most fundamental linguistic units for communication. Auditory sentence comprehension, which engages nearly all core processes involved in language comprehension [1], is frequently impaired in individuals with post-stroke aphasia and often remains compromised even after auditory word comprehension has recovered [2]. Post-stroke auditory sentence comprehension deficits may arise from various underlying causes, including syntactic processing impairments, cognitive control deficits [3,4], and working memory impairments [5].

Various neuroimaging techniques have been employed to underpin the neural correlates of auditory sentence comprehension, including functional magnetic resonance imaging (fMRI) [6,7,8], voxel-based lesion symptom mapping (VLSM) [9,10,11,12,13,14,15,16], structural disconnection mapping [16,17], connectome-based lesion-symptom mapping (CLSM), and tract- [18,19,20] or connectome-level analyses [10,13,15,21] of diffusion magnetic resonance imaging (dMRI). Based on evidence from these studies, current neurocognitive models of sentence processing [1,3,22,23,24] describe a network of left perisylvian brain regions that are central to auditory sentence comprehension, with particular emphasis on left temporal areas. However, the specific contribution of left frontal regions—particularly the left posterior inferior frontal gyrus—remains debated. Some models suggest it is crucial for core syntactic computations and complex sentence processing [3,22], while others argue that its role is either non-specific or primarily confined to sentence production rather than comprehension [24]. As for white matter, dorsal tracts have been considered crucial for auditory sentence comprehension [1,22,25,26]. More specifically, the left arcuate fasciculus (AF) and superior longitudinal fasciculus (SLF) have been implicated in syntactic processing [26,27,28,29], phonological processing [30], and verbal working memory [15]. Evidence also suggests that ventral tracts contribute to sentence comprehension [13,15,24]. For example, the left inferior longitudinal fasciculus (ILF) and inferior fronto-occipital fasciculus (IFOF) are associated with syntactic processing [15] and general sentence comprehension [16,29]. The left extreme capsule fiber system, which is considered a continuous ventral fiber system encompassing uncinate fasciculus and IFOF [31,32], has also been implicated in supporting syntactic processing [27,33,34]. In addition, white matter tracts in the right hemisphere [35] and interhemispheric tracts—particularly the corpus callosum [15,22,36,37]—have also been suggested to be important for auditory sentence comprehension.

However, previous neuroimaging studies have primarily been conducted at the voxel level, limiting the capacity to accurately resolve complex fiber architectures, such as crossing fibers. Recently, a sub-voxel resolution neuroimaging technique called fixel-based analysis (FBA) has been developed. FBA is an advanced diffusion MRI (dMRI) analytical framework that enables statistical inference at the level of individual fiber populations within voxels—termed fixels. FBA can estimate multiple fiber orientations per voxel [38,39], offering a more precise and detailed representation of white matter structure, particularly in regions with fiber crossings. FBA employs three fixel-derived metrics: fiber density (FD) [40], fiber cross-section (FC), and their combined measure, fiber density and cross-section (FDC) [41]. FD reflects the intra-axonal volume fraction at the microstructural level, whereas FC indicates the macroscopic cross-sectional area of fiber bundles. Consequently, FDC, the product of FD and FC, relates to the total intra-axonal volume, serving as an index of information transmission capacity. This approach enhances sensitivity and interpretability when identifying microstructural and morphological alterations along specific fiber tracts, thus overcoming the limitations inherent to voxel-averaged measures, which can be confounded by complex fiber architectures [41].

Previous research on the neural basis of auditory sentence comprehension has primarily focused on English-speaking participants, with few studies including Mandarin-speaking aphasic patients. Evidence from Mandarin speakers could enhance the understanding of whether specific brain structures support sentence comprehension universally across languages.

A common pattern seen in patients with aphasia is difficulty with sentences that deviate from the typical word order for a language. In English and Chinese, this is the Subject-Verb-Object order, which corresponds to the Agent-Theme order. Canonical sentences (Example 1a–c) follow this typical word order. Non-canonical sentences deviate from this standard word order by moving constituents such as the object across the verb and subject. Ba-sentences (Example 2a) in Mandarin Chinese are sentences in which the object is placed immediately after the function word ‘把’ (ba), followed by the verb or verb phrase, resulting in non-canonical Subject-Object-Verb order. Passive sentences (Example b) are typical non-canonical sentences both in Chinese and English, as they follow the Theme-Agent order. Object extracted wh-questions (Example 2c) are considered non-canonical in Mandarin Chinese because wh-words move to the sentence initial position at Logical Form [42]. In Mandarin Chinese, subject relative clauses (Example 2d) are also non-canonical because they reverse the Agent-Theme order, unlike English where object relative clauses are non-canonical. Despite structural differences, both languages show increased difficulty with non-canonical sentences [43].

**Example** **1.** ***Example of canonical sentences in Mandarin Chinese**. **(a)** active sentence; **(b)** subject-extracted wh-question; **(c)** object relative clause.***(a)** Mandarin*男人**在**埋葬**女人**[nanren**zai**maizang**nüren]*manPROGburywoman‘ The man is burying the woman. ’**(b)** Mandarin*谁**在**埋葬**女人？**[shui**zai**maizang**nüren]*whoPROGburywoman‘ Who is burying the woman. ’**(c)** Mandarin*男人**埋葬**的**女人**戴着**帽子 。**[nanren**maizang**de**nüren**dai-zhe**maozi]*manburyRELwomanwear-PROGhat‘The woman who the man buries is wearing a hat.’

**Example** **2.** ***Example of non-canonical sentences in Mandarin Chinese**. **(a)** ba-sentence; **(b)** passive sentence; **(c)** object extracted wh-question; **(d)** subject relative clause.***(a)** Mandarin*女人**把**男人**埋葬 了 。**[nüren**ba**nanren**maizang-le]*womanBAmanbury-PFV‘ The woman buried the man. ’**(b)** Mandarin*女人**被**男人*埋葬了。*[nüren**bei**nanren*maizang-le]womanBEImanbury-PFV‘ The woman was buried by the man. ’**(c)** Mandarin*男人**在**埋葬**谁 ？**[nanren**zai**maizang**shui]*manPROGburywho‘ Who is the man burying? ’**(d)** Mandarin*埋葬**男人**的**女人**戴着**帽子 。**[maizang**nanren**de**nüren**dai-zhe**maozi]*burymanRELwomanwear-PROGhat‘The woman who buries the man is wearing a hat.’

This study aims to precisely map white matter structures associated with auditory sentence comprehension impairments in 23 native Mandarin-speaking individuals with aphasia during the subacute stage of stroke using FBA. We focus on the subacute stage to minimize the influence of non-linguistic factors—such as neurological instability and consciousness disturbance—on the accurate assessment of language function in the acute stage, and to reduce the influence of extensive neural plasticity and functional reorganization typically observed in the chronic stage.

## 2. Materials and Methods

### 2.1. Participants

Participants in this study were from an ongoing post-stroke aphasia cohort at the Department of Rehabilitation, Huashan Hospital, Fudan University, recruiting from inpatients or regular outpatients receiving rehabilitation treatment. The present analysis utilized data from all patients enrolled between July 2024 and February 2025. Inclusion criteria for the cohort comprised a confirmed diagnosis of a single-hemispheric stroke within six months before the assessment and subsequent aphasia verified by the Mandarin version of the Western Aphasia Battery (MAB). Exclusion criteria encompassed comorbid neurological disorders (e.g., moyamoya disease, multiple sclerosis, Parkinson’s disease, and other Parkinsonian syndromes), psychiatric disorders (e.g., major depressive disorder, bipolar disorder, schizophrenia), and contraindications to magnetic resonance imaging. To ensure the absence of significant non-linguistic cognitive impairments, patients with Non-Language-based Cognitive Assessment (NLCA) scores ≤ 65 were excluded. A total of 385 stroke patients without aphasia or with comorbidities were excluded from the cohort, as were 107 patients with post-stroke aphasia exhibiting severe non-language cognitive deficits. Ultimately, 23 participants (19 men and 4 women) were included in the current analysis, with a mean stroke duration of 9.4 weeks at assessment. All participants were native Mandarin speakers, right-handed (based on Edinburgh Handedness Inventory), and had normal or corrected-to-normal hearing and vision. Among the 23 participants, 11 were diagnosed with Broca’s aphasia, 8 with anomic aphasia, 2 with Wernicke’s aphasia, and 2 with transcortical motor aphasia, according to the MAB. All participants provided informed consent according to the protocol approved by the institutional review board. Demographic details are summarized in Table 1.

### 2.2. Behavioral Data

Participants’ non-linguistic cognitive abilities were assessed using the Non-language-based Cognitive Assessment (NLCA) [44], which evaluates five non-linguistic cognitive domains: visuospatial function, attention, memory, reasoning, and executive function. The maximum total score is 80.

The Mandarin version of the Western Aphasia Battery (MAB) [45] was administered to evaluate the overall severity of aphasia in participants. Adapted from the verbal section of the original Western Aphasia Battery, the MAB comprises ten subtests: Conversational Questions, Picture Description, Yes/No Questions, Auditory Word Recognition, Sequential Commands, Repetition, Object Naming, Word Fluency, Sentence Completion, and Responsive Speech. The Aphasia Quotient (AQ), a weighted average of the subtest scores, serves as an index of overall aphasia severity.

The Assessment of Verbs and Sentences from the Chinese Aphasia Language Battery (CALB-AVS) [46], adapted from the Northwestern Assessment of Verbs and Sentences (NAVS) [47], was used to evaluate verb and sentence deficits in the participants. The CALB-AVS comprises five subtests, among which the Verb Comprehension Test and the Sentence Comprehension Test were the focus of this study. The Verb Comprehension Test consists of 20 trials in which participants were asked to identify the correct image from four action pictures within five seconds after hearing a verb. In the Sentence Comprehension Test, participants were asked to match an auditorily presented sentence to the corresponding picture, choosing between two role-reversed alternatives. The 28 test sentences represent seven sentence types, three canonical—active sentences (Example 1a), subject-extracted wh-questions (Example 1b), object relative clauses (Example 1c); and four non-canonical—ba-sentences (Example 2a), passive sentences (Example 2b), object-extracted wh-questions (Example 2c), and subject relative clauses (Example 2d).

### 2.3. Image Acquisition

dMRI images were acquired using a 3.0T uMR 790 scanner (United Imaging Healthcare, Shanghai, China) with an echo planar imaging (EPI) sequence (time of repetition [TR] = 3835 ms; time of echo [TE] = 75.7 ms; flip angle = 90°; field of view [FOV] = 209 × 209 mm^2^; matrix size = 116 × 116; number of slices = 78; voxel resolution = 1.8 × 1.8 × 1.8 mm^3^; multiband factor = 3; acquisition time = 6 min 38 s). A total of 92 non-collinear diffusion-weighted directions were collected at b-values of 1500 s/mm^2^ and 3000 s/mm^2^, along with six interleaved b = 0 s/mm^2^ images. All images were acquired with both anterior–posterior (AP) and posterior–anterior (PA) phase-encoding directions for susceptibility distortion correction.

T1-weighted (T1w), T2-weighted (T2w), and fluid-attenuated inversion recovery (FLAIR) images were also acquired at the same MRI session using the same scanner as part of the protocol of the aphasia cohort. T1w images were acquired with a fast spoiled gradient echo sequence (TR = 7.2 ms; TE = 2.7 ms; flip angle = 8°; FOV = 208 × 300 mm^2^; matrix size = 116 × 116; number of slices = 320; voxel resolution = 0.8 × 0.8 × 0.8 mm^3^). T1w images were used for lesion segmentation, while T2w and FLAIR images were not used in the current study. The total scan time for all images was approximately 35 min.

### 2.4. Image Preprocessing

dMRI data were preprocessed using MRtrix3 (version 3.0.4) [48] and FSL (version 6.0.7.9) [49]. A brief overview of the preprocessing pipeline is provided below. First, the data were denoised using MRtrix3’s dwidenoise [50], followed by removal of Gibbs ringing artifacts [51]. Susceptibility-induced off-resonance fields were estimated using TOPUP from FSL [52]. Subsequently, eddy current-induced distortion correction, susceptibility-induced distortion correction [53], outlier detection and replacement [54], between-volumes and within-volumes motion correction [55] as well as susceptibility-by-movement correction [56] were performed with EDDY from FSL. B_1_ field inhomogeneity was corrected using the N4 algorithm [57]. The preprocessed dMRI data were resampled to ACPC orientation, and their corresponding gradient directions were rotated accordingly. Finally, brain extraction was performed using SynthStrip [58]. All preprocessing steps were visually inspected for quality control

### 2.5. Lesion Segmentation

**Figure 1:**
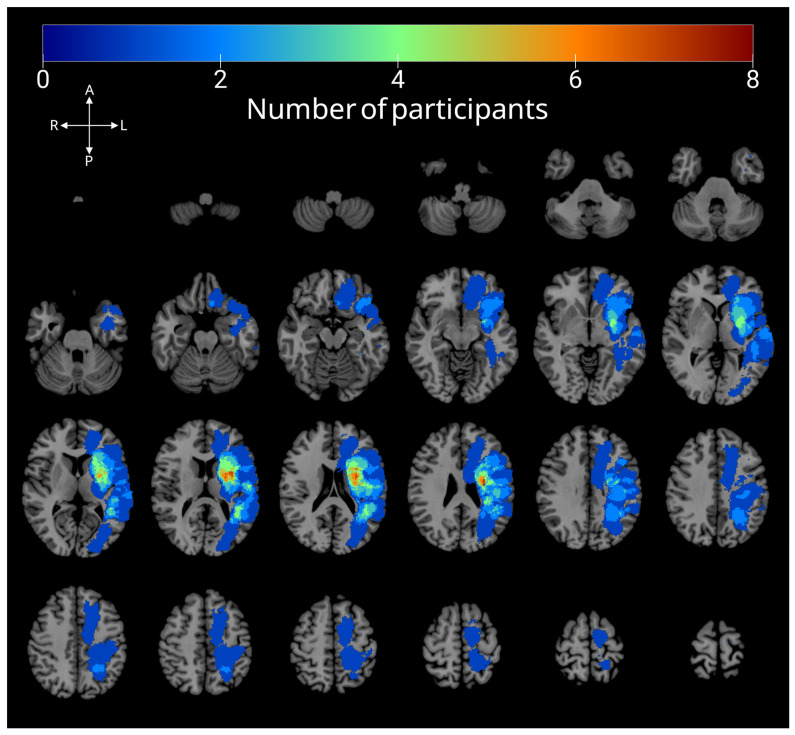
Lesion overlay map for all participants. Color represents the number of participants with lesions at each voxel.

Stroke lesions were automatically segmented from participants’ T1w images and spatially normalized to the Colin27 template using the LINDA (Lesion Identification with Neighborhood Data Analysis) package [59] in R. The lesion masks were then manually checked and, if necessary, edited to correct errors in the automatic segmentation. Figure 1 shows the lesion overlay map.

### 2.6. Fixel-Based Analyses (FBA)

FBA analyses were conducted using MRtrix3 (version 3.0.4) [48]. Following preprocessing, tissue response functions were estimated [60,61] and averaged across participants. The dMRI data and brain masks were subsequently upsampled to an isotropic voxel size of 1.25 mm. Fiber orientation distributions (FODs) were estimated using multi-tissue spherical deconvolution with group-averaged tissue response functions [39]. Global intensity normalization of FODs was performed using mtnormalise [62]. A study-specific FOD template was generated, and each subject’s FOD data was registered to this template [63]. The template FODs were then segmented into fixels [64], producing a template fixel mask for subsequent fixel-based analyses. Metrics including FD, FC, and FDC were calculated. FC values were log-transformed [log(FC)] to achieve normal distribution centered around zero. Whole-brain fiber tractography [65] was performed on the FOD template, followed by Spherical-deconvolution Informed Filtering of Tractograms (SIFT) [64] to reduce tractography biases. A fixel-fixel connectivity matrix was then generated based on the SIFT-filtered whole brain tractogram to facilitate fixel smoothing and connectivity-based fixel enhancement [66,67]. Finally, the FD, log(FC), and FDC data underwent connectivity-based spatial smoothing, where smoothing weights were calculated by multiplying a 10mm full-width half-maximum (FWHM) Gaussian kernel with the fixel–fixel connectivity weights [66].

### 2.7. Tract Segmentation

Tract segmentation was performed using TractSeg (version 2.9) [68]. Peaks of the spherical harmonic function were extracted from each voxel in the template FOD image [69]. These peaks were input into TractSeg to generate tract masks, tract endpoint regions, and tract orientation maps (TOMs) [70]. For all 72 tracts defined in TractSeg [68,71], probabilistic tractography was conducted on TOMs. Only fibers not leaving the bundle mask and starting and ending in the endpoint regions were kept [72]. Each tract’s resulting tractogram comprised 2000 streamlines. Corresponding fixel tract density maps [73] were then generated and binarized to create fixel masks for each tract.

### 2.8. Statistical Analyses

Behavioral analyses were conducted using R (version 4.5.1; R core team 2025). Non-parametric statistical analyses were performed due to violations of normality assumptions. A Wilcoxon signed-rank test was used to compare comprehension accuracy between canonical sentences and non-canonical sentences. Then the Friedman test, a non-parametric alternative to repeated measures ANOVA, was employed to assess overall differences in comprehension accuracy across multiple sentence types within subjects. Upon finding a significant main effect, post hoc pairwise comparisons were performed using paired Wilcoxon signed-rank tests with Bonferroni-Holm correction to control for multiple testing, identifying specific differences between sentence types. Spearman correlation analyses were conducted to examine the relationships among AQ, auditory word recognition scores, verb comprehension accuracy, and comprehension accuracy for canonical sentences and non-canonical sentences.

Whole-brain fixel-based analyses were performed using MRtrix3 (version 3.0.4). Linear regression models were fit with fixel-wise FDC as the response variable and accuracy for non-canonical sentences from the Sentence Comprehension Test of the CALB-AVS as the predictor. Covariates include age, years of education, log-transformed time post-stroke, and the overall accuracy of the Verb Comprehension Test from the CALB-AVS. Following established guidance for controlling false positives in FBA [74], our primary analyses focus exclusively on FDC. However, we also conducted post hoc exploratory analyses with analogous models for FD and log(FC) to investigate the contributions of fiber microstructure and morphometry. All variables in these models were mean-centered and scaled to unit variance. Correlation coefficients of the predictors of interest served as effect size measures. Connectivity-based fixel enhancement [66,67] was applied to enhance the statistical maps. Family-wise error (FWE)-corrected *p*-values were computed using nonparametric bootstrapping with 5000 permutations, and statistical significance was set at an FWE-corrected threshold of *p* < 0.05. The current study was conducted among stroke survivors, whose lesions could lead to outliers in fixel measures in the corresponding regions. To assess the robustness of our primary analysis results against potential bias introduced by outliers in FDC (the response variable in our primary analyses), we reanalyzed the data using the same linear regression models while excluding outliers identified by Cook’s distance ≥ 1 [75].

Tract-wise analyses were performed using R (version 4.5.1; R core team 2025). Tracts containing > 0.1% significant fixel identified in the FBA in their fixel masks were selected as tracts of interest. Partial Spearman’s correlations were performed between mean FDC values within these tracts of interest and the accuracy scores from each sentence type of the Sentence Comprehension Test from the CALB-AVS, with age, years of education, log-transformed time post-stroke, and accuracy scores of the Verb Comprehension Test from the CALB-AVS as covariates. Analogous post hoc exploratory correlation analyses were conducted for mean FD and mean log(FC). Statistical significance was defined using a Bonferroni-Holm-corrected threshold of *p* < 0.05.

## 3. Results

### 3.1. Behavioral Analyses

**Figure 2:**
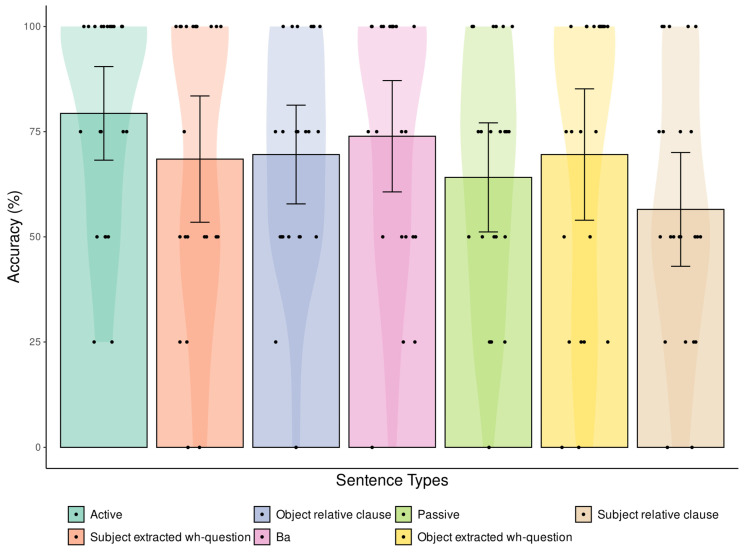
Comprehension accuracy of different sentence types. Shown are means (histogram), 95% confidence intervals of the mean (bars) and data distributions (violin plots) of the accuracy for each sentence type in the Sentence Comprehension Test from the CALB-AVS. Each data point represents individual participant performance. Only subject relative clauses showed significantly decreased accuracy compared to active sentences.

The means, 95% confidence intervals of the mean, and data distribution of accuracy for each sentence type in the Sentence Comprehension Test from the CALB-AVS are presented in Figure 2. Comprehension accuracy for non-canonical sentences was significantly lower than for canonical sentences (*p* = 0.010). Specifically, participants performed significantly worse on subject relative clauses compared to active sentences (Bonferroni-Holm-corrected *p* = 0.003). No other significant differences in comprehension accuracy were observed among the remaining sentence types.

**Figure 3:**
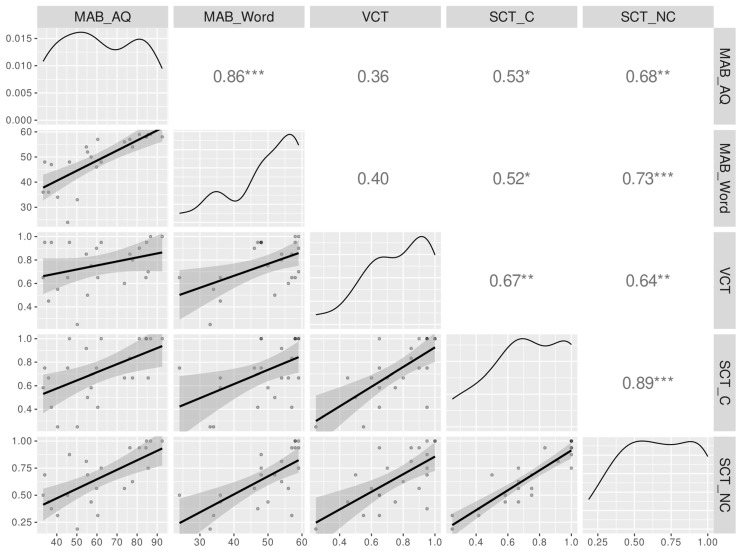
Correlational analyses of selected language assessment results. (**Upper triangle**): Spearman correlation coefficients between variables, with asterisks indicating the Bonferroni-Holm-corrected *p*-value levels: * = *p* < 0.05, ** = *p* < 0.01, *** = *p* < 0.001. (**Diagonal**): density plots of variables. (**Lower triangle**): scatter plots of variables with LOESS smooth lines and shaded confidence intervals. Abbreviations: MAB_AQ = Aphasia Quotient from the Mandarin version of the Western Aphasia Battery (MAB); MAB_word = Auditory word recognition score from MAB; VCT = Overall accuracy on the Verb Comprehension Test; SCT_C = Accuracy of canonical sentence comprehension in the Sentence Comprehension Test; SCT_NC = Accuracy of non-canonical sentence comprehension in the Sentence Comprehension Test.

Correlational analyses (Figure 3) demonstrated a strong positive association between comprehension accuracy for canonical vs. non-canonical sentences (r_s_ = 0.89, Bonferroni-Holm-corrected *p* < 0.001). Additionally, comprehension accuracy for canonical sentences was positively correlated with verb comprehension accuracy (r_s_ = 0.67, Bonferroni-Holm-corrected *p* < 0.01), auditory word recognition score (r_s_ = 0.52, Bonferroni-Holm-corrected *p* < 0.05) and AQ (r_s_ = 0.53, Bonferroni-Holm-corrected *p* < 0.05). Similarly, comprehension accuracy for non-canonical sentences showed positive correlations not only with verb comprehension accuracy (r_s_ = 0.64, Bonferroni-Holm-corrected *p* < 0.01) but also with auditory word recognition score (r_s_ = 0.73, Bonferroni-Holm-corrected *p* < 0.001) and AQ (r_s_ = 0.68, Bonferroni-Holm-corrected *p* < 0.01).

### 3.2. Fixel-Based Analyses

**Figure 4:**
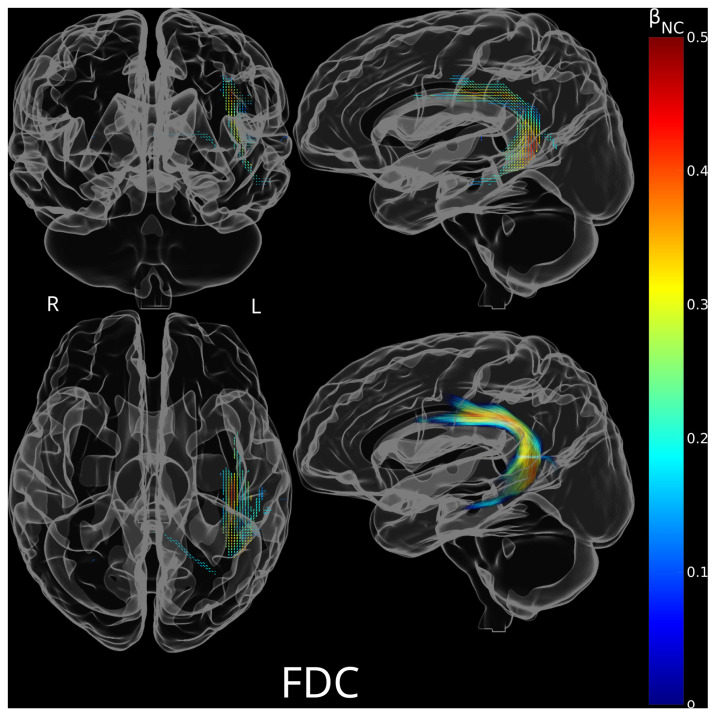
Significant fixels for positive association between non-canonical sentence comprehension accuracy and fiber density and cross-section product (FDC), colored by the standardized regression coefficients of non-canonical sentence comprehension accuracy (β_NC_) in the linear models. A total of 1601 significant fixels were found. primarily located in the left perisylvian region and the posterior corpus callosum. (**Top left**): Coronal view of significant fixels. (**Top right**): Sagittal view of significant fixels. (**Bottom left**): Axial view of significant fixels. (**Bottom right**): Sagittal view of streamlines traversing significant fixels. Abbreviations: R = Right; L = Left.

**Figure A1:**
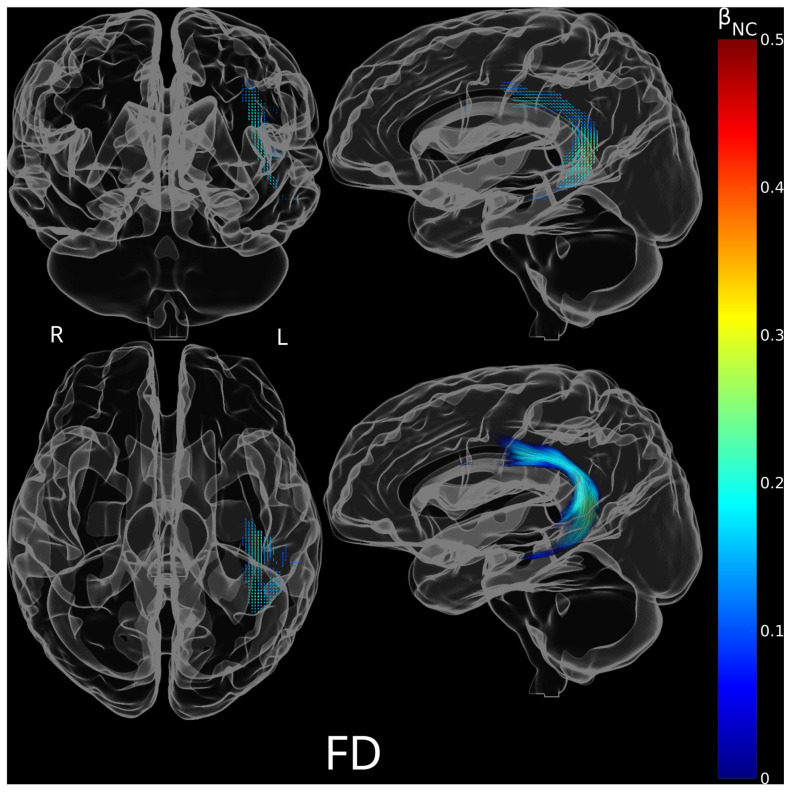
Significant fixels for positive association between non-canonical sentence comprehension accuracy and fiber density (FD), colored by the standardized regression coefficients of non-canonical sentence comprehension accuracy (β_NC_) in the linear models. A total of 1003 significant fixels were found, primarily located in the left perisylvian region. (**Top left**): Coronal view of significant fixels. (**Top right**): Sagittal view of significant fixels. (**Bottom left**): Axial view of significant fixels. (**Bottom right**): Sagittal view of streamlines traversing significant fixels. Abbreviations: R = Right; L = Left.

**Figure A2:**
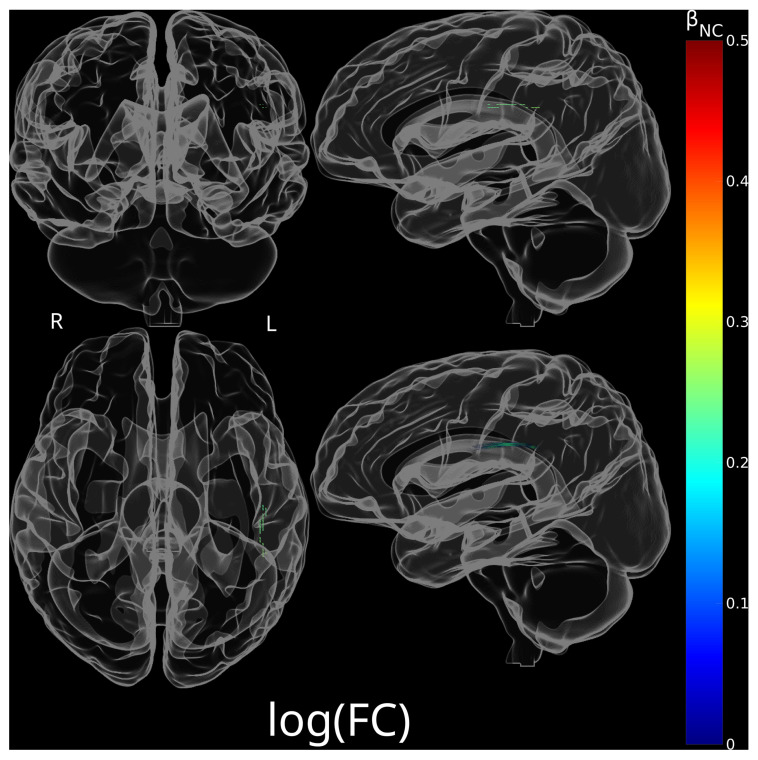
Significant fixels for positive association between non-canonical sentence comprehension accuracy and log-transformed fiber cross-section [log(FC)], colored by the standardized regression coefficients of non-canonical sentence comprehension accuracy (β_NC_) in the linear models. A total of 20 significant fixels were found. primarily located in the left parietal region. (**Top left**): Coronal view of significant fixels. (**Top right**): Sagittal view of significant fixels. (**Bottom left**): Axial view of significant fixels. (**Bottom right**): Sagittal view of streamlines traversing significant fixels. Abbreviations: R = Right; L = Left.

Figure 4 illustrates the spatial distribution of 1601 fixels where FDC exhibited a significant positive association with non-canonical sentence comprehension accuracy (family-wise error corrected *p* < 0.05), controlling for age, education, log-transformed time post stroke, and verb comprehension accuracy. These fixels were predominantly situated in the left perisylvian region and the posterior corpus callosum. No fixel demonstrated a negative relationship between FDC and non-canonical sentence comprehension accuracy. The results of the same analysis after excluding outliers are shown in Figure A7, which revealed a similar pattern. Post hoc exploratory analyses revealed a broader perisylvian distribution of fixels (1003 fixels) in which impaired non-canonical sentence comprehension was linked to reduced FD (Figure A1), compared to a more restricted parietal distribution of fixels (20 fixels) where impaired non-canonical sentence comprehension was associated with reduced FC (Figure A2).

**Figure 5:**
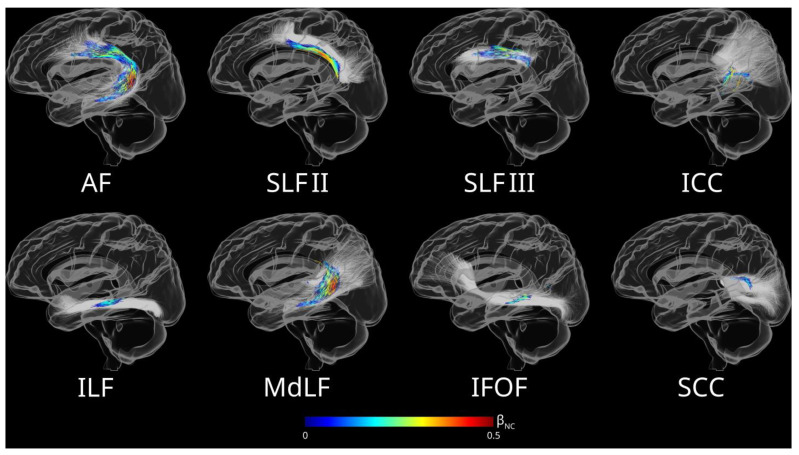
Streamlines illustrating the spatial relationship between fixels, where FDC was significantly associated with non-canonical sentence comprehension accuracy after controlling for covariates, and white matter tracts. Streamlines are colored based on effect size (the standardized regression coefficients of non-canonical sentence comprehension accuracy from the corresponding linear models).

Tracts that contained significant fixels exhibiting associations between reduced FDC and impaired non-canonical sentence comprehension are listed in Table 2. A positive linear relationship was observed between non-canonical sentence comprehension accuracy and FDC in fixels located in the left superior longitudinal fasciculus II (SLF II), superior longitudinal fasciculus III (SLF III), arcuate fasciculus (AF), middle longitudinal fasciculus (MdLF), inferior fronto-occipital fasciculus (IFOF), inferior longitudinal fasciculus (ILF), and the isthmus (ICC) and splenium (SCC) of the corpus callosum. Figure 5 illustrates the spatial distribution of significant fixels relative to white matter tracts. Specifically, significant fixels were primarily located in the ventral portion of the left SLF II, the caudal portion of the left SLF III, the middle portion of the left IFOF, the left ILF, the left MdLF, the rostral-ventral portion of the ICC, and the rostral-dorsal portion of the SCC.

**Table 2:** White matter tracts containing significant fixels where FDC was positively associated with non-canonical sentence comprehension accuracy (family-wise error corrected *p* < 0.05).

Tract *	Number of Significant Fixels in Tract	Number of Total Fixels in Tract	Percentage of Significant Fixels in Tract	Max Effect Size (β_NC_) †
AF left	1522	22,478	6.77%	0.47
SLF III left	763	7873	9.69%	0.47
MdLF left	581	23,711	2.45%	0.43
SLF II left	350	11,695	2.99%	0.42
ICC	218	44,676	0.49%	0.41
ST_PAR left	67	27,839	0.24%	0.40
ILF left	43	7001	0.61%	0.24
IFOF left	40	15,602	0.26%	0.31
SCC	38	18,847	0.20%	0.20
ST_OCC left	4	11,730	0.03%	0.30
ST_POSTC left	3	11,850	0.03%	0.21
SLF I left	2	10,757	0.02%	0.19
ST_PREC left	2	15,527	0.01%	0.20
CST left	1	10,985	0.01%	0.19
T_PREC left	1	14,438	0.01%	0.19

* AF = arcuate fasciculus; SLF III = superior longitudinal fasciculus III; MdLF = middle longitudinal fasciculus; SLF II = superior longitudinal fasciculus II; ICC = isthmus of the corpus callosum; ST_PAR = striato-parietal tract; ILF = inferior longitudinal fasciculus; IFOF = inferior fronto-occipital fasciculus; SCC = splenium of corpus callosum; ST_OCC = striato-occipital tract; ST_POSTC = striato-postcentral tract; SLF I = superior longitudinal fasciculus I; ST_PREC = striato-precentral; CST = corticospinal tract; T_PREC = thalamo-precentral tract. † β_NC_ = standardized regression coefficients of non-canonical sentence comprehension accuracy in the linear models.

**Figure A3:**
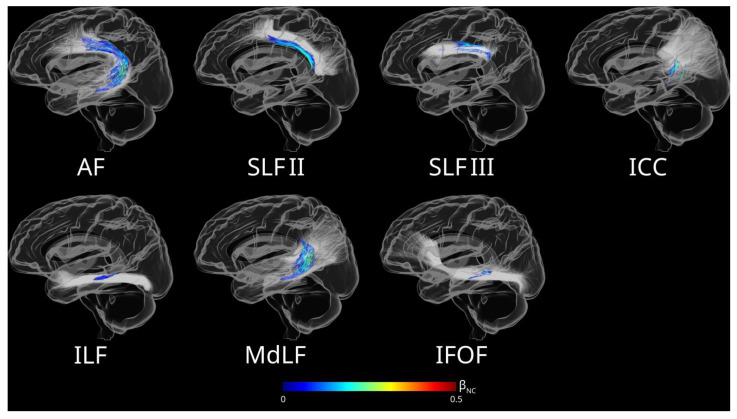
Streamlines illustrating the special relationship between fixels, where FD was significantly associated with non-canonical sentence comprehension accuracy after controlling for covariates, and white matter tracts. Streamlines are colored based on effect size (the standardized regression coefficients of non-canonical sentence comprehension accuracy from the corresponding linear models).

**Figure A4:**
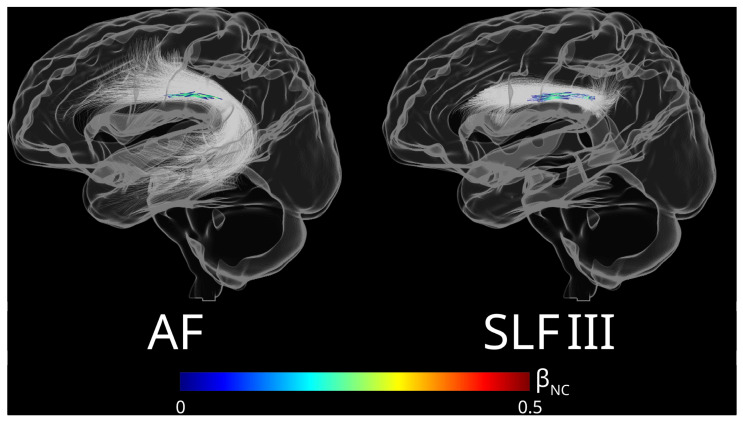
Streamlines illustrating the special relationship between fixels, where FC was significantly associated with non-canonical sentence comprehension accuracy after controlling for covariates, and white matter tracts. Streamlines are colored based on effect size (the standardized regression coefficients of non-canonical sentence comprehension accuracy from the corresponding linear models).

Exploratory analyses showed fixels where FD reductions associated with impaired comprehension of non-canonical sentences had a similar spatial distribution pattern as that of FDC (Figure A3). In contrast, only fixels located in the caudal portion of the left SLF III and caudal parietal portion of the left AF exhibited FC reductions associated with impaired comprehension of non-canonical sentences (Figure A4).

### 3.3. Tract-Wise Analyses

**Figure 6:**
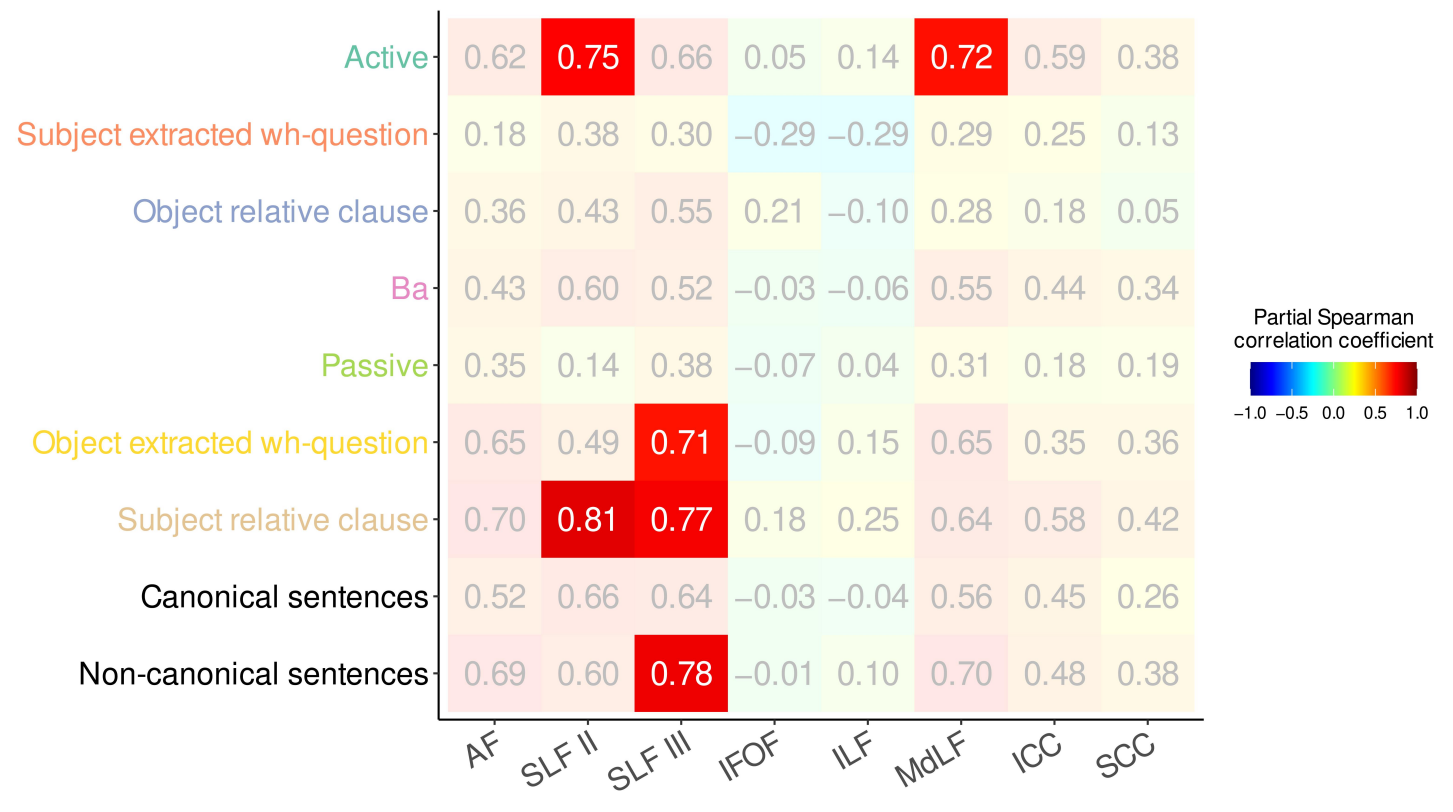
Partial Spearman correlation coefficients between comprehension accuracy for different sentence types and mean FDC of various tracts of interest, controlling for age, education, log-transformed time post stroke and verb comprehension accuracy. Cells indicating correlations that survived Bonferroni-Holm correction for multiple comparisons (family-wise α = 0.05, adjusted α = 0.000694) are opaque with white labels; cells not surviving the correction are displayed with reduced opacity and gray labels.

Tract-wise partial correlation analyses revealed that comprehension accuracy for active sentences was positively associated with the mean FDC of the left MdLF and SLF II (Figure 6). Accuracy in comprehending object extracted sentences, subject relative clauses, and all non-canonical sentences was positively correlated with the mean FDC of the left SLF III. Additionally, comprehension accuracy for subject relative clauses was also positively associated with the mean FDC of the left SLF II.

**Figure A5:**
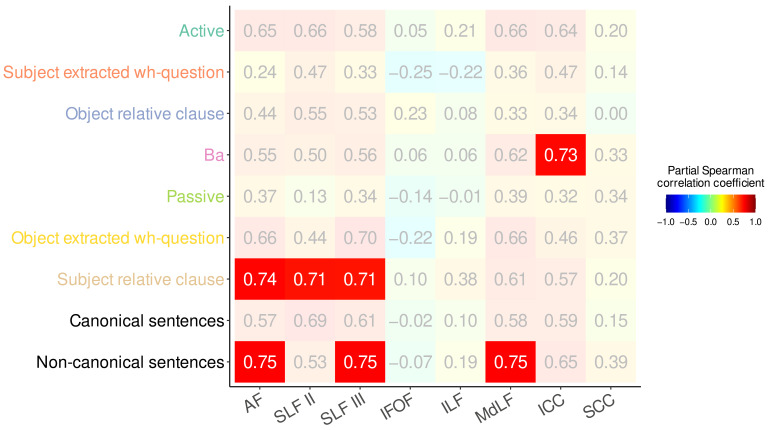
Partial Spearman correlation coefficients between comprehension accuracy for different sentence types and mean FD of various tracts of interest, controlling for age, education, log-transformed time post stroke and verb comprehension accuracy. Cells indicating correlations that survived Bonferroni-Holm correction for multiple comparisons (family-wise α = 0.05, adjusted α = 0.000694) are opaque with white labels; cells not surviving the correction are displayed with reduced opacity and gray labels.

**Figure A7:**
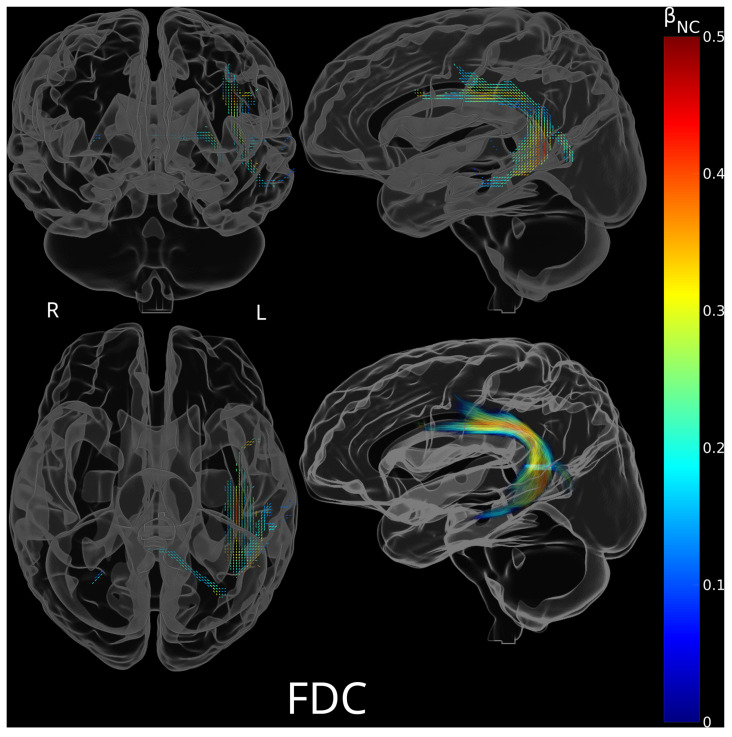
Significant fixels for positive association between non-canonical sentence comprehension accuracy and fiber density and cross-section product (FDC), after removing outliers using Cook’s distance. Fixels are colored by the standardized regression coefficients of non-canonical sentence comprehension accuracy (β_NC_) in the linear models. A total of 2052 significant fixels were found. primarily located in the left perisylvian region and the posterior corpus callosum. Notice that significant fixels extended slightly laterally in the left frontal and temporal regions as well as slightly rightward in the posterior corpus callosum, compared to Figure 4. (**Top left**): Coronal view of significant fixels. (**Top right**): Sagittal view of significant fixels. (**Bottom left**): Axial view of significant fixels. (**Bottom right**): Sagittal view of streamlines traversing significant fixels. Abbreviations: R = Right; L = Left.

Regarding FD (Figure A5), comprehension accuracy for ba-sentences was positively correlated with the mean FD of the ICC, while comprehension accuracy for subject relative clauses was positively associated with the mean FD of the left AF, left SLF II, and left SLF III. Comprehension accuracy for non-canonical sentences was positively correlated with the mean FD of the left AF, left SLF III, and left MdLF.

No statistically significant correlation was found between comprehension accuracy for any sentence type and the mean FC of the tracts of interest.

## 4. Discussion

### 4.1. Behavioral Analysis Results

The sentence comprehension test results showed poorer performance on non-canonical sentences compared to canonical sentences in Mandarin speakers with post-stroke aphasia, consistent with findings in native English speakers with aphasia [47]. Comprehension of non-canonical sentences requires assigning thematic roles in an order that deviates from the typical "agent-verb-patient/theme" sequence, thereby demanding greater processing resources [76], which are likely compromised in aphasic patients. Among the sentence types examined, subject relative clauses exhibited the greatest impairment. In Mandarin, successful comprehension of subject relative clauses depends not only on the ability to assign thematic roles (similar to object relative clauses in English) but also on hierarchical structure processing and sufficient working memory capacity [77]. These increased demands likely make subject relative clauses more vulnerable in aphasia.

### 4.2. Fixel-Based Analysis Results

This study aimed to identify the white matter pathways underlying auditory comprehension of sentences. Our results indicated that reduced FDC in the left peri-Sylvian white matter was associated with impaired comprehension of non-canonical sentences. The significant fixels likely corresponded to the left SLF II, SLF III, AF, IFOF, ILF, MdLF, ICC, and SCC. Exploratory analyses indicated that FDC reduction within these tracts was primarily driven by FD reduction, suggesting microstructural axon loss or damage [41]. Only the left SLF III and AF exhibited FC reduction, indicative of macrostructural tract atrophy [41]. Reduced FD has been suggested to reflect a decreased number of axons or reduced intra-axonal size within the fiber tract, while FC initially remains unchanged as extra-axonal space is filled by inflammatory or glial cells. Subsequent clearance of debris leads to fiber tract atrophy and reduced FC [41]. Therefore, FD reductions may signify early fiber damage, whereas the observed FC reduction in the left SLF III and AF may indicate more advanced neurodegeneration and atrophy. These findings are consistent with previous fixel-based studies in stroke patients demonstrating widespread FD reduction accompanied by more localized FC reduction [78,79]. A detailed discussion of each white matter tracts’ role in sentence comprehension follows.

#### 4.2.1. Dorsal Language Stream Tracts (AF, SLF)

Our findings in Mandarin speakers are in line with previous works in English speakers suggesting that complex sentence comprehension depends on left dorsal tracts [1,25,26,28], supporting a language-general role of the left AF and SLF in auditory processing of syntactically complex sentences.

The AF connects the temporal lobe to the inferior and middle frontal gyri as well as the precentral gyrus of the same hemisphere [68,71]. The anatomical connections of AF allow for efficient communication between temporal regions (processing auditory input and meaning) and frontal regions (involved in syntactic processing and working memory). According to Friederici’s theory [22], the left AF plays a crucial role in the neural network underlying sentence comprehension by supporting the integration and processing of syntactic information. Damage to the left AF, particularly its long segment (connecting the temporal and the frontal areas) [20,80], has been associated with impaired understanding of sentences [81], especially syntactically complex sentences such as object extraction ones [16].

The SLF II connects the inferior parietal lobule (notably the angular gyrus) and the lateral parieto-occipital cortex to the dorsolateral prefrontal cortex (including the middle and superior frontal gyri) [68,71,82]. The left SLF II is thought to support the integration of information necessary for understanding complex sentence structures, likely by facilitating communication between regions responsible for higher-level syntactic operations and working memory [83]. The disconnection of left SLF has been shown to impair comprehension of complex sentences [16].

The SLF III, the ventral branch of the SLF, connects the supramarginal gyrus—which is thought to contribute to phonological working memory and the integration of semantic and syntactic information [22,84,85]—to the inferior frontal gyrus [68,71,82,86], which is involved in syntactic processing, semantic processing, and executive control [22,87,88]. This tract and its cortical endings constitute an important part of the fronto-parietal network [89], supporting semantic prediction and integration in sentence comprehension [90]. The lesion to the left SLF III has been found to be associated with impaired semantic memory [91] and comprehension of complex sentences [16].

#### 4.2.2. Ventral Language Stream Tracts (IFOF, ILF, MdLF)

Ventral stream tracts such as IFOF and ILF have been proposed to be part of the system for processing receptive syntax [15,24]. The IFOF connects the orbitofrontal and inferior frontal gyri to the ipsilateral occipital lobe [68,71], whereas the ILF links the ventral and lateral temporal cortices with the occipital lobe [68,71]. Evidence indicates that the left IFOF supports semantic processing [92] and is broadly involved in various language functions, including sentence comprehension [29]. The contribution of the left ILF to sentence comprehension likely stems from its role in enabling lexical-semantic mapping [93], which in turn facilitates more efficient semantic processing [94]. The lesion of the left IFOF and ILF has been associated with sentence comprehension deficits in people with aphasia [15,16,95], consistent with the findings of the present study.

The MdLF, as defined in TractSeg [68,71], is a long association fiber tract connecting the superior temporal gyrus and temporal pole to the parietal lobe. Although the role of MdLF in language remains relatively understudied [93], evidence suggests its involvement in semantic processing [96], attention and verbal working memory during speech processing [97]. Damage to the left MdLF has been associated with impaired auditory comprehension of both canonical and non-canonical sentences [15,16].

#### 4.2.3. Beyond Traditional Dual-Stream Tracts

The ICC contains fibers that interconnect the posterior parietal and superior temporal cortices across hemispheres [68,71]. It plays a critical role in integrating information processed separately by the left and right hemispheres during auditory language comprehension. The left hemisphere is typically specialized for syntactic and lexical-semantic processing, while the right hemisphere is more involved in processing prosodic (intonation, rhythm) and other suprasegmental features [1]. Patients with lesions in the posterior corpus callosum often exhibit impaired coordination between syntactic and prosodic cues during auditory sentence comprehension, despite preserved basic syntactic processing [37]. Consistent with our current findings, previous studies using indirect structural disconnection mapping [16] and connectome-based lesion-symptom mapping [13,15] have implicated the posterior corpus callosum in sentence comprehension.

### 4.3. Tract-Wise Analysis Results

It is worth highlighting that our tract-wise analyses have further demonstrated relationships between FDC reduction in white matter tracts and auditory comprehension impairments of different sentence types. Due to the relatively small sample size of this study, it is possible that not all relationships were revealed. However, current results already indicated that FDC reduction in the left MdLF was associated with impaired comprehension of simple active sentences, which is in line with previous findings [16], further supporting MdLF’s involvement in general sentence comprehension. Additionally, FDC reduction in the left SLF II was associated with impaired comprehension of subject relative clauses, reinforcing its role in supporting the comprehension of complex sentence structures. Similarly, FDC reduction in the left SLF III correlated with impaired comprehension of object extracted wh-questions and subject relative clauses, both requiring the maintenance and integration of displaced logical [42] or syntactic components, thus suggesting the role of the left SLF III in working memory [15,16].

### 4.4. Research and Clinical Implications

Our results support the theory that the dorsal pathway (including left AF and SLF) is involved in the processing of syntactically complex sentences and that the posterior part of the corpus callosum facilitates interplay between syntactic and prosodic information, as proposed in Friederici’s model of the neural basis of language comprehension [1,23]. Our findings not only provide cross-linguistic evidence for the current model, but also offer insight into the specific spatial distribution of white matter fibers involved in the model.

Clinically, these insights have important implications for improving language ability in patients with post-stroke aphasia. Transcranial magnetic stimulation (TMS) and other noninvasive brain stimulation techniques have demonstrated efficacy in improving language ability for post-stroke aphasia in addition to speech and language therapy [98,99,100]. However, the optimal stimulation sites and parameters to enhance sentence comprehension remain unclear. Recent evidence suggests that TMS is more likely to activate white matter than gray matter [101]. Precise localization of white matter that is related to sentence comprehension deficits could enable personalized tractography-based navigated TMS [102,103], potentially improving therapeutic effects. Moreover, white matter morphological measures of fiber tracts identified in this study could serve as biomarkers to assess the neuroplastic effects of therapies targeting sentence comprehension.

### 4.5. Limitations

The present study has several limitations. First, the relatively small sample size of this study may reduce its statistical power. Previous studies employing fixel-based analysis have shown reasonable results with a similar sample size of 20–30 subjects [104,105,106,107]. Several tools have been developed for power analysis of voxel-based neuroimaging study [108,109], but there is still no dedicated tool for power analysis of fixel-based study. Therefore, a post hoc power analysis was conducted for FBA using R package pwr [110] (see Figure A for the results). Results of the post hoc power analysis indicated that the sample size of 23 was enough for detecting significant fixels in the left peri-sylvian region and the corpus callosum. The relatively small sample size did restrict sub-group analysis for different types of aphasia. Second, deficits in non-canonical sentence comprehension may arise from different underlying causes, such as problems in syntactic processing, deficits in cognitive control [4], or limitations in verbal working memory [15]. Despite that, we used NLCA to exclude severe cognitive deficits but did not capture subtler impairments in working memory or attention. This study did not delineate among these potential contributing factors. Future studies may benefit from adding verbal working memory tasks to control for these factors. Third, neighboring white matter fixel masks may overlap, meaning that a single fixel could be associated with multiple white matter tracts. Thus, the associations between fixel location and tracts are not definite. The number and percentage of significant fixels overlapping with another tract are shown in Table A1. The fiber bundles that include a large proportion of overlapping fixels in the significant fixel are: left SLF II, left SLF III, left MdLF, left ILF, left IFOF, and SCC. Tract-wise results in these tracts should be taken with caution. Finally, this study was cross-sectional and focused on subacute stroke patients, which complicates direct comparison with previous studies primarily conducted on chronic stroke populations. Reduced fractional anisotropy (FA), mean diffusivity (MD), axial diffusivity (AD), and radial diffusivity (RD) have been observed in lesional white matter during the subacute stage compared to the chronic stage of stroke, possibly resulting from the presence of cellular debris, inflammatory infiltration and relatively preserved myelin in the lesional area during the subacute phase [111]. It is reasonable to speculate that similar effects would influence fixel-based metrics. Longitudinal studies extending into the chronic phase might help in addressing this problem.

### 4.6. Future Directions

Future research should incorporate verbal working memory tasks to distinguish white matter tracts involved in verbal working memory from those related to complex syntax processing. Furthermore, structural MRI analyses can be integrated to provide complementary information on gray matter regions associated with auditory sentence comprehension and clarify the spatial relationships between white and gray matter correlates of sentence comprehension. With a larger sample size, subgroup analyses of aphasia types can be performed to elucidate distinct neural correlates. Subsequent longitudinal studies will provide more evidence regarding the relationship between post-stroke white matter plasticity and sentence auditory comprehension ability.

## 5. Conclusions

Fixel-based analysis revealed that reduced FDC of fixels in the left AF, SLF II, SLF III, IFOF, ILF, MdLF, ICC, and SCC is related to impaired non-canonical sentence comprehension. Tract-wise analyses revealed dissociative associations between distinct tracts and varying levels of syntactic complexity. These findings provide novel insights that can inform future research and refine current neurocognitive models of sentence comprehension.

## Figures and Tables

**Table 1:** Participants’ demographic and behavioral data.

Variables	Range, Mean and Standard Deviation
Age (years)	Range 30–79; M = 55.4; SD = 12.6
Education (years of formal schooling)	Range 6–19; M = 13.5; SD = 3.9
Time post stroke (weeks)	Range 4–20; M = 9.4; SD = 4.7
Total score of the NLCA	Range 68–79; M = 73.9; SD = 3.2
Aphasia Quotient from the MAB	Range 33.2–92.5; M = 61.5; SD = 19.3
Score on the Auditory Word Recognition task of the MAB	Range 24–59; M = 49.2; SD = 10.1
Accuracy of the Verb Comprehension Test in the CALB-AVS (%)	Range 25–100; M = 75.9; SD = 20.2
Accuracy of canonical sentences in the Sentence Comprehension Test of the CALB-AVS (%)	Range 25–100; M = 72.5; SD = 24.5
Accuracy of non-canonical sentences in the Sentence Comprehension Test of the CALB-AVS (%)	Range 18.8–100; M = 66.0; SD = 25.1

NLCA = Non-language-based Cognitive Assessment; MAB = Mandarin version of the Western Aphasia Battery; CALB-AVS = Assessment of Verbs and Sentences from the Chinese Aphasia Language Battery.

**Figure A6:**
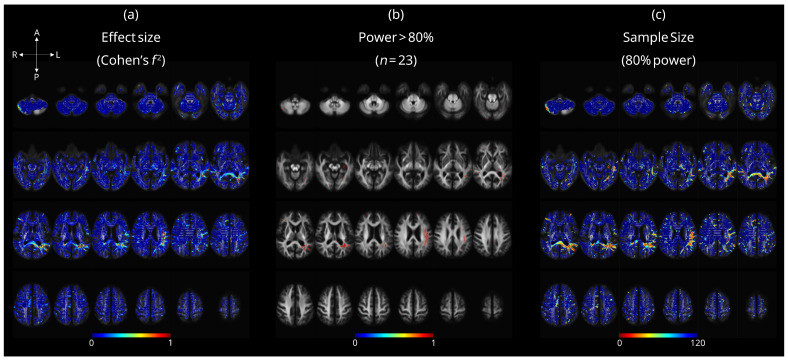
Results of post hoc power analysis. (**a**) Effect size (Cohen’s *f*^2^) map for the primary FBA. (**b**) Based on the effect size map, a power map was generated for *n* = 23 subjects. (**c**) The corresponding sample size map was also generated to determine the number of subjects required with 80% power.

## Data Availability

Due to privacy regulations, the clinical data collected in this study are not deposited in a public registry, but the data can be made available via a request to the corresponding author. Anonymized data can be made available after the approval of the participants and when a signed data transfer agreement is in place.

## Abbreviations

The following abbreviations are used in this manuscript:

dMRI	Diffusion magnetic resonance imaging
fMRI	Functional magnetic resonance imaging
VLSM	Voxel-based lesion symptom mapping
CLSM	Connectome-based lesion-symptom mapping
FBA	Fixel-based analysis
FOD	Fiber orientation distribution
FD	Fiber density
FC	Fiber cross-section
Log(FC)	Log-transformed fiber cross-section
FDC	Product of fiber density and cross-section
MAB	Mandarin version of the Western Aphasia Battery
AQ	Aphasia quotient
NLCA	Non-language-based Cognitive Assessment
NAVS	Northwestern Assessment of Verbs and Sentences
CALB-AVS	Assessment of Verbs and Sentences from the Chinese Aphasia Language Battery
EPI	Echo planar imaging
TR	Time of repetition
TE	Time of echo
FOV	Field of view
TOM	Tract orientation maps
FWE	Family-wise error
SIFT	Spherical-deconvolution Informed Filtering of Tractograms
SLF II	Superior longitudinal fasciculus II
SLF III	Superior longitudinal fasciculus III
AF	Arcuate fasciculus
MdLF	Middle longitudinal fasciculus
IFOF	Inferior fronto-occipital fasciculus
ILF	Inferior longitudinal fasciculus
ICC	Isthmus of the corpus callosum
SCC	Splenium of corpus callosum

## Appendix A

**Table A1:** Significant fixels overlapping between tracts.

Number of Overlapping Significant Fixels	Tract 1 *	Number of Significant Fixels in Tract 1	Percentage of Significant Fixels in Tract 1 that Overlap	Tract 2 *	Number of Significant Fixels in Tract 2	Percentage of Significant Fixels in Tract 2 that Overlap
38	SCC	38	100%	ICC	218	17%
43	ILF left	43	100%	MdLF left	581	7%
579	MdLF left	581	100%	AF left	1522	38%
347	SLF II left	350	99%	AF left	1522	23%
42	ILF left	43	98%	AF left	1522	3%
39	IFOF left	40	98%	MdLF left	581	7%
39	IFOF left	40	98%	AF left	1522	3%
743	SLF III left	763	97%	AF left	1522	49%
180	ICC	218	83%	AF left	1522	12%
153	ICC	218	70%	MdLF left	581	26%
21	IFOF left	40	53%	ILF left	43	49%
156	SLF II left	350	45%	SLF III left	763	20%
6	IFOF left	40	15%	ICC	218	3%
45	SLF II left	350	13%	MdLF left	581	8%
49	MdLF left	581	8%	SLF III left	763	6%
1	SCC	38	3%	IFOF left	40	3%
1	ILF left	43	2%	ICC	218	0%
3	ICC	218	1%	SLF II left	350	1%

* AF = arcuate fasciculus; ICC = isthmus of the corpus callosum; IFOF = inferior fronto-occipital fasciculus; ILF = inferior longitudinal fasciculus; MdLF = middle longitudinal fasciculus; SCC = splenium of corpus callosum; SLF II = superior longitudinal fasciculus II; SLF III = superior longitudinal fasciculus III.
